# A Data-Driven Framework for Small Hydroelectric Plant Prognosis Using Tsfresh and Machine Learning Survival Models

**DOI:** 10.3390/s23010012

**Published:** 2022-12-20

**Authors:** Rodrigo Barbosa de Santis, Tiago Silveira Gontijo, Marcelo Azevedo Costa

**Affiliations:** 1 Graduate Program in Industrial Engineering, Universidade Federal de Minas Gerais, Av. Antônio Carlos 6627, Belo Horizonte 31270-901, MG, Brazil; 2 Department of Industrial Engineering, Universidade Federal de Minas Gerais, Av. Antônio Carlos 6627, Belo Horizonte 31270-901, MG, Brazil

**Keywords:** hydroelectric power plant, condition-based maintenance, prognosis, survival analysis, time series feature engineering, survival random forest

## Abstract

Maintenance in small hydroelectric plants (SHPs) is essential for securing the expansion of clean energy sources and supplying the energy estimated to be required for the coming years. Identifying failures in SHPs before they happen is crucial for allowing better management of asset maintenance, lowering operating costs, and enabling the expansion of renewable energy sources. Most fault prognosis models proposed thus far for hydroelectric generating units are based on signal decomposition and regression models. In the specific case of SHPs, there is a high occurrence of data being censored, since the operation is not consistently steady and can be repeatedly interrupted due to transmission problems or scarcity of water resources. To overcome this, we propose a two-step, data-driven framework for SHP prognosis based on time series feature engineering and survival modeling. We compared two different strategies for feature engineering: one using higher-order statistics and the other using the Tsfresh algorithm. We adjusted three machine learning survival models—CoxNet, survival random forests, and gradient boosting survival analysis—for estimating the concordance index of these approaches. The best model presented a significant concordance index of 77.44%. We further investigated and discussed the importance of the monitored sensors and the feature extraction aggregations. The kurtosis and variance were the most relevant aggregations in the higher-order statistics domain, while the fast Fourier transform and continuous wavelet transform were the most frequent transformations when using Tsfresh. The most important sensors were related to the temperature at several points, such as the bearing generator, oil hydraulic unit, and turbine radial bushing.

## 1. Introduction

The expansion of renewable energy sources is vital for ensuring the energy supply of a fast-paced market growing in the coming decades, with expectations for it to double by 2060 [1]. Clean energy already accounts for three quarters of newly installed capacity annually [1], and those related to water resources are the most-used ones. The building of small hydroelectric plants (SHPs), which accounts for a significant share of this group, has increased worldwide due to the lower initial investment, lower operating costs, and expanding regulation of energy markets. The potential total energy generation capacity of these SHPs is twice the total capacity of the currently installed energy plants [2].

The maintenance of a hydropower plant is a complex task. It demands a specific level of skill to ensure an adequate level of dependability of the asset through its useful life. There are three kinds of maintenance. The first and most basic is corrective maintenance, in which a component is replaced after a failure occurs. The second is preventive maintenance, which estimates the service life of a component and realizes a replacement once the operating lifetime is reached. Finally, there is predictive maintenance, in which the system condition is assessed from data periodically or continually acquired from various sensors [3,4]. A predictive or condition-based maintenance system consists of two stages. The first stage is the diagnosis, which incorporates fault detection or anomalous operating conditions, fault isolation by subcomponents, and identification of the character and degree of the failure [4]. The second stage is the prognosis, which involves using statistical and machine learning models in order to calculate the use life of the assets and the confidence interval of the estimation [5], foresee maintenance, and increase the dependability and availability of the generation units.

Many data-driven models have been proposed for fault detection and diagnosis in hydroelectric plants. These models include principal component analysis (PCA) [6], independent component analysis (ICA) [7], and a least square support vector machine [8,9,10,11]. PCA decomposition is used to assist specialists in determining and selecting the principal features which contribute to cavitation in hydro-turbines [12]. Current studies have presented a new monitoring method based on ICA-PCA that can extract both non-Gaussian and Gaussian information from operating data for fault detection and diagnosis [13]. This ICA-PCA method has been expanded with the adoption of a nonlinear kernel transformation prior to the application of the decomposition method, which has become known as kernel ICA-PCA [14]. Zhu et al. applied this method in the hydropower generation context with increased success rates and lower fault detection delays than either the PCA or ICA-PCA applications. While most models rely on signal processing, De Souza Gomes et al. proposed functional analysis and computational intelligence models for fault classification in power transmission lines [15]. Santis and Costa proposed the application of isolation iForest for small hydroelectric monitoring, where iForest isolates anomalous sensor readings by creating a health index based on the average distance of the points to the tree root [16]. Hara et al. extended iForest’s performance by implementing a preliminary step of feature selection using the Hilbert–Schmidt independence criterion [17]. It is worth emphasizing that in addition to data-driven models, there is the application of analytic model-based methods, which have been presenting significant design results in the context of fault diagnosis in power systems, such as in [18].

For prognoses, the techniques generally applied for estimating the use life are classified into statistical techniques, comprising regression techniques [19], Wiener-, Gamma-, and Markovian-based processes such as machine learning techniques, comprising neural networks, vector support machines, and electrical signature analysis [20], and principal component analysis [21], as well as deep learning techniques more recently, comprising auto-encoder, recurrent, and convolutional neural networks [22].

Reports on the prognoses of hydroelectric generating units are scarcer than publications related to their diagnosis [23]. A great challenge in the area is proposing procedures that contemplate faults between different generating units and auxiliary interconnected systems [23]. An et al. presented a prognosis model based on the application of Shepard’s interpolation of three variables: bearing vibration, apparent power, and working head [24]. The signal is decomposed by applying intrinsic time-scale decomposition to a limited number of rotating components, and the artificial neural network is trained for each of the temporal components of the signal. Thereafter, the models present a similar framework, with varied individual methods for signal decomposition and regression models. Fu et al. applied variational mode decomposition for signal decomposition and a least square support vector machine regression model fine-tuned using an adaptive sine cosine algorithm [8,25]. Zhou et al. combined a feature strategy using empirical wavelet decomposition for decomposing and Gram–Schmidt orthogonal process feature selection combined with kernel extreme learning machine regression [26].

Since feature extraction is a key factor in the success of data-driven diagnosis and prognosis systems, the Time Series Feature Extraction Based on Scalable Hypothesis Tests (TSFRESH, or TSF for short) algorithm has gained prominent attention in the literature, leading to better results than physical and statistical features alone [27]. The algorithm is capable of generating hundreds of new features while reducing collinearity through its hypothesis test-integrated selection procedure. Tan et al. adopted TSF along with a probability-based forest for bearing diagnosis [28]. A two-stage feature learning approach combining TSF and a multi-layer perceptron classifier was adopted for anomaly detection in machinery processes by Tnani et al. [44] and for earthquake detection by Khan et al. [29].

Finally, the random survival forest (RSF) is a survival analysis model that has recently been adapted for data-driven maintenance prognosis systems. Voronov et al. proposed the application of RSF for heavy vehicle battery prognosis [30], an important part of the electrical system and mostly affected by lead-acid during the engine starting. Gurung adopted the RSF along with histogram data for interpretive modeling and prediction of the remaining survival time of components of heavy-duty trucks, aiming to improve operation and maintenance processes [31]. Snider and McBean proposed an RSF-based model for the water main pipe replacement model, expecting savings of USD 26 million, or 14% [32] of the total cost of the ductile iron pipe, over the next 50 years.

In this context, the present paper innovates by proposing a framework for the prognosis of hydroelectric plants, based on the TSF feature extraction and selection algorithm and survival analysis models. The authors did not find any evidence or study that has adopted a similar approach in the literature thus far. We compare the different strategies of feature engineering associated with three survival model analyses, evaluating the models using the concordance index metric. The main findings and contributions of the current paper are the following:

- The proposal of a data-oriented framework including feature engineering strategies and machine learning survival models for intelligent fault diagnosis of the SHP generating unit;
- Evaluation of the importance of attributes using the permutation importance method associated with the RSF survival model;
- Affirmation that the RSF survival analysis model associated with the TSF feature engineering hybrid model obtained the highest concordance index score (77.44%).

The remainder of the present article is organized as follows. Section 2 defines the study methodology, describing the methods, algorithms, and dataset applied. Section 3 presents the results and discussions of the simulations of the models, in addition to the outputs of the feature engineering strategies and survival analysis models, with illustrative examples of those models’ inference. Finally, Section 4 presents the conclusions and recommendations for future work.

## 2. Problem Formulation

The prognosis problem was formulated as an inference problem based on historical data, specialist knowledge, external factors, and future usage profiles. Prognosis is a condition-based maintenance (CBM) practice widely applied to reduce costs incurred during inefficient schedule-based maintenance. In mechanical systems, the repetitive stresses from rotating machinery vibration temperature cycles leads to structural failures. Since mechanical parts commonly move slowly to a critical level, monitoring the growth of these failures permits evaluating the degradation and estimating the remaining component life over a period of time [33].

The current study was developed in Ado Popinhak, an SHP situated in the southern region of Brazil. With an installed capacity of 22.6 MW, the plant supplies energy to 50,000 residences. Monitoring data from the main single hydro generator unit were registered every 5 min, and the study period was from 13 August 2018 to 9 August 2019. Table 1 describes the number of runs by the generators contained in the dataset, the number of runs that ended due to failure, the average cycle time per run, and the longest cycle time.

The objective was to predict the remaining useful life (RUL) of a power system based on multiple component-level sensor and event data. The RUL information allows decision makers to better plan maintenance and interventions, improving availability and reducing costs.

**Figure 1:**
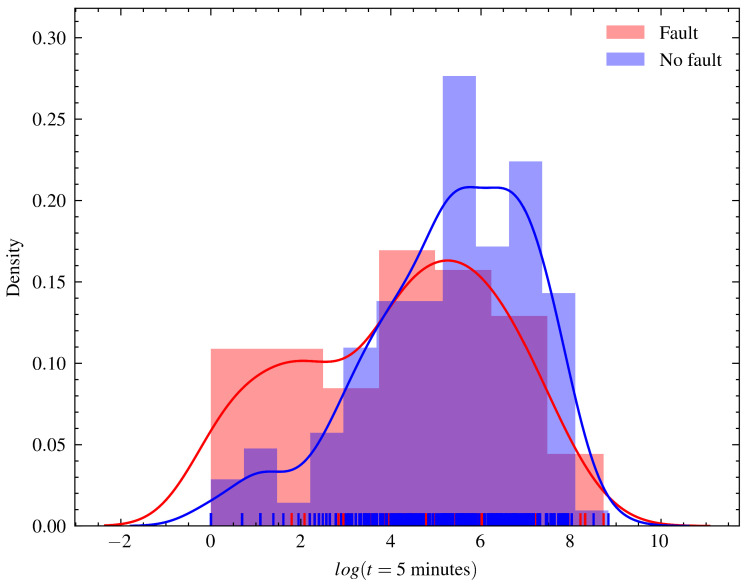
Distribution chart of the last operating cycle time registered for runs with and without faults. The time cycle scale was converted to the logarithmic scale in order to better show the distribution of the variable. The average maximum cycle time of failed runs is less than that of normal operations. The distribution of both presents a bimodal characteristic, with two different concentration points more clearly verified in the faulty series.

Raw sensor reading data and event data, such as interventions, shutdowns, and planned and corrective maintenance, were curated and merged. The data registered were classified and split into runs, which are periods from the moment the generating unit is turned on until it is shut down, whether due to failure or not. Runs that ended because of failure were labeled with a true or false failure label. The time distribution plot until the end of the runs that ended in failure and those that were interrupted for another reason is shown in Figure 1.

The nature of the problem is interpreted as a problem of survival analysis, given that the system does not run to failure and can be shut down due to a lack of water resources for generation, failures in the transmission system, or the execution of scheduled maintenance. A summary of the characteristics of the problem and dataset is as follows:

- Data were collected for four generator units of the same manufacturer, model, and age;
- Fifty-four variables were monitored, and readings were registered in the transactional database every 5 min;
- Data were heterogeneous, including either control settings or monitored variables, both numerical and categorical;
- Missing data represented around 5% of total registrations, mostly caused by loss of a network connection between the remote plants and the operations center;
- The runs in which the subsequent state was a forced stop were labeled, and the last reading was registered as the logged time of failure;
- There were many runs where no failure was registered during the time (i.e., data were right-censored).

**Figure 2:**
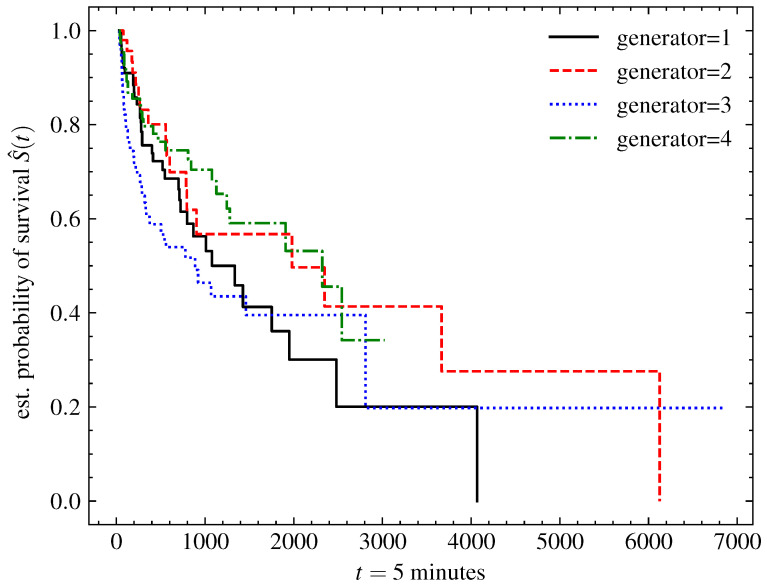
Kaplan–Meier model adjusted for each of the generators. The decay rate of the survival function of the adjusted model for each of the generators was similar, indicating the correlated behavior of the health of the generating units.

The runs were considered independent, given that the systems could be turned off for a long time and undergo modifications, such as routine maintenance, during this period, and because machine start-up is the biggest cause of system deterioration. For this reason, the Kaplan–Meier model was adjusted and presented in order to describe the survival function of each run of the four generators in Figure 2.

**Figure 3:**
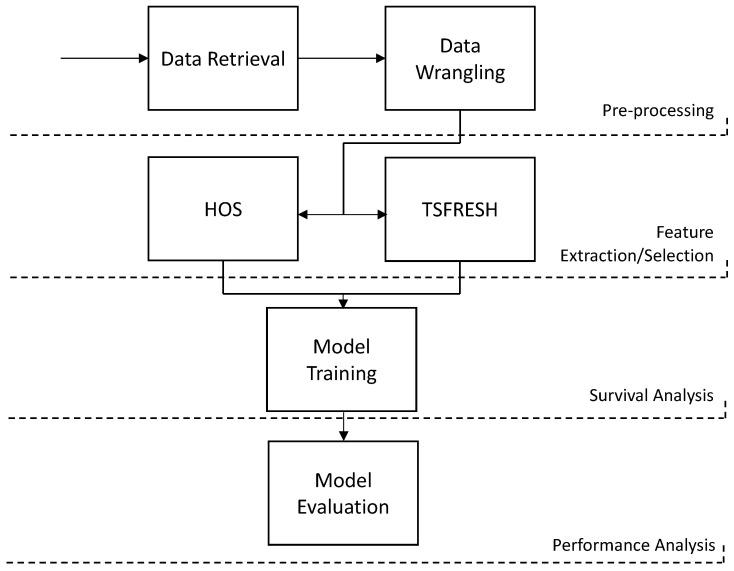
Data transformation flow from data retrieval to performance analysis.

The data transformation workflow is described in Figure 3. Sensor and event data were collected from the transactional database of telemetric systems and stored in text files. In the data-wrangling phase, the record tables were parsed and joined with the event tables, and the records were resampled into 5 min periods. While still in this stage, the imputation of the missing data and the classification of the runs were made if they ended due to failure or programmed shutdowns (censoring).

In the feature extraction and selection step, the features were extracted from each of the time series of the sensors during the first 30 5 min time units (150 min of operation) using the feature engineering strategies described in Section 3.1. The fixed period of the first 30 cycle times was selected from each run to extract features and adjust the survival models. Runs with a cycle time of fewer than 30 s were excluded from the training base. This approach was adopted to avoid data leakage in model training, where size-related features can contribute to models readily predicting the estimated total time to failure. These features were recorded in a text file and zipped due to the size of the generated tables.

In the next step, the runs were randomly divided into training and test sets, using proportions of 90% of the runs for training and 10% for testing. In each of the simulations, the partitioning was performed using a different random seed. The models were fitted to the training set, and metrics were calculated on the training set. The computational time was calculated for each fit of the models and saved for later analysis.

Finally, the model metrics were compared using a set of statistical tests in order to identify if there was a difference in the average scores for different groups of models or feature strategies.

## 3. Materials and Methods

### 3.1. Time Series Feature Engineering

#### 3.1.1. Higher-Order Statistics (HOS)

Higher-order statistics (HOS) have been applied in different fields which require separation and characterization of non-Gaussian signals against a Gaussian background. Moments and cumulants are widely used to quantify certain probability distributions, such as location (first moment) and scale (second moment). Several authors have used HOS in signal processing. For example, De La Rosa and Muñoz reported the application of higher-order cumulants via signal processing using HOS for early detection of subterranean termites [34], while Nemer et al. presented an algorithm for robust voice activity based on third- and fourth-order cumulants of speech [35].

Let X=[x(t)],t=0,1,2,3,… be a real stationary discrete-time signal and its moments up to order *p* exist. Then, its *p*th-order moment can be given by [35,36]

(1) $$m_{p}\left(\tau_{1},\tau_{2},\ldots ,\tau_{p-1}\right)\equiv E\left\{x\left(t\right)x\left(t+\tau_{1}\right)\ldots x\left(t+\tau_{p-1}\right)\right\}$$

depending solely on the time differences $\tau_{1},\tau_{2},\ldots ,\tau_{p-1}$ for all *i*. E(.) represents the statistical expectation for a deterministic signal. If the signal has zero mean as well, then its cumulant functions are given by [35]

(2) $$\text{second-order}\mathrm{cumulant}:C_{2}\left(\tau_{1}\right)=m_{2}\left(\tau_{1}\right)$$

(3) $$\text{third-order}\mathrm{cumulant}:C_{2}\left(\tau_{1},\tau_{2}\right)=m_{3}\left(\tau_{1},\tau_{2}\right)$$

(4) $$\begin{array}{c} \text{fourth-order}\mathrm{cumulant}:C_{4}\left(\tau_{1},\tau_{2},\tau_{3}\right)=m_{4}\left(\tau_{1},\tau_{2},\tau_{3}\right)-\\ m_{2}\left(\tau_{1}\right)*m_{2}\left(\tau_{3}-\tau_{2}\right)-m_{2}\left(\tau_{2}\right).m_{2}\left(\tau_{3}-\tau_{1}\right)-m_{2}\left(\tau_{3}\right).m_{2}\left(\tau_{2}-\tau_{1}\right) \end{array}$$

By setting all the lags to zero in the cumulant expressions and normalizing the input data to have a unity variance, we obtained the variance, normalized skewness, and normalized kurtosis:

(5) $$\mathrm{variance}:\gamma_{2}\equiv C_{2}\left(0\right)=E\left\{x^{2}\left(n\right)\right\}$$

(6) $$\mathrm{normalized}\mathrm{skewness}:\gamma_{3}\equiv \frac{C_{3}\left(0,0\right)}{{\left[C_{2}\left(0\right)\right]}^{1.5}}=\frac{E\{x^{3}\left(n\right)\}}{{\left[E\left\{x^{2}\left(n\right)\right\}\right]}^{1.5}}$$

(7) $$\mathrm{normalized}\mathrm{kurtosis}:\gamma_{4}\equiv \frac{C_{4}\left(0,0,0\right)}{{\left[C_{2}\left(0\right)\right]}^{2}}=\frac{E\{x^{4}\left(n\right)\}}{{\left[E\left\{x^{2}\left(n\right)\right\}\right]}^{2}}$$

The skewness indicates to which side of the distribution the data are concentrated for unimodal distributions, so a positive skew indicates that the tail is to the right, and a negative skew indicates that it is to the left. The kurtosis is usually associated with the measure of the “peakedness” of the probability distribution of a real-valued random variable. Higher kurtosis means that more of the variance is due to infrequent extreme deviations, as opposed to frequent, modestly sized deviations. The first four moments were calculated for each of the runs in order to extract the basic descriptive variables of the sensor signals:

#### 3.1.2. Tsfresh (TSF)

TSF is an algorithm presented for time series feature engineering, which accelerates this procedure by combining 63 time series characterization methods. Features are chosen based on automatically configured hypotheses [37].

Given a set of time series $D={\left\{X_{i}\right\}}_{i=1}^{N}$, each time series $X_{i}$ is mapped into a feature space with a problem-specific dimensionality *M* and feature vector ${\vec{x}}_{i}=\left(x_{i,1},x_{i,w},\ldots ,x_{i,M}\right)$. The feature vector ${\vec{x}}_{i}$ is built by applying time series characterization methods $f_{j}:X_{i}\to x_{i,j}$ to the respective time series $X_{i}$, which results in the feature vector [37]

(8) $${\vec{x}}_{i}=(f_{1}\left(X_{i}\right),f_{2}\left(X_{i}\right),\ldots ,f_{M}\left(X_{i}\right)$$

The feature vector might be extended by additional univariate attributes $\{a_{i,1},a_{i,2},\ldots ,$$a_{i,U}{\}}_{i=1}^{N}$ and feature vectors from other kinds of time series. For a machine learning system with *K* different time series and *U* univariate variables per sample *i*, the resulting design matrix would have *i* rows and (K·M+U) columns [37].

From the set of 63 characterization methods $f_{j}$ available in the algorithm, we illustrate two of the most important ones based on our feature analysis, which are the fast Fourier transform (FFT) and the continuous wavelet transform (CWT). Both methods are time–frequency decomposition methods often applied in signal analysis.

The discrete Fourier transform (DFT) is a signal decomposition technique adequate for discrete and periodic signals. Let a signal $a_{n}$ for n=0,…,N−1 and $a_{n}=a_{n+jN}$ for all *n* and *j*. The discrete Fourier transform of *a*, also known as the spectrum of *a*, is described by [38]

(9) $$A_{k}=\sum_{n=0}^{N-1}W_{N}^{kn}a_{n}$$

where $W_{N}=e^{-i\frac{2\pi }{N}}$ and $W_{N}^{k}$ are called the *N*th roots of unity. The sequence $A_{k}$ is the DFT of the sequence $a_{n}$, where each is a sequence of *N* complex numbers. The FFT is a fast algorithm for computing the DFT into $log_{2}N$ states, each of which consists of fewer computations [38].

The CWT of a signal *a* with the wavelet ψ is defined as [39]

(10) $$W_{\psi }a\left(s,t\right)=\frac{1}{\sqrt{s}}\int_{-\infty }^{+\infty }a\left(x\right)\psi \frac{t-x}{s}dx$$

where the scale *s* is inversely proportional to the central frequency of the rescaled wavelet $\psi_{s}\left(x\right)=\psi x/s$, which is a bandpass, and *t* represents the time location of the signal analysis. The larger the scale *s*, the wider the analyzing function ψ(x), and therefore the smaller the corresponding examined frequency. The main advantage over the Fourier transform methods is that the frequency description is localized in time, and that window size varies. It gives more flexibility and effectiveness than fixed-size analysis since low frequencies can be analyzed over wide time windows, while high frequencies can be analyzed over narrow time windows [39].

Finally, the feature selection of TSF is used to filter out irrelevant features based on automated statistical hypothesis tests [37]. Feature selection is crucial to reducing the number of variables, which increases generalization and prevents overfitting, in addition to bringing speed gains and less complexity to the estimator [40].

### 3.2. Survival Analysis

#### 3.2.1. Evaluation Metrics

The most employed evaluation metric of survival models is the concordance index (C-index or C-statistic) [41]. It reflects a model’s capacity of ranking the survival times based on the individual risk scores, and it can be expressed by the formula [42]

(11) $$\text{C-index}=\frac{\sum_{i,j}1_{T_{j}<T_{i}}\cdot 1_{\eta_{j}>\eta_{i}}\cdot \delta_{j}}{\sum_{i,j}1_{T_{j}<T_{i}}\cdot \delta_{j}}$$

where $\eta_{i}$ is the risk score of a unit, $1_{T_{j}<T_{i}}=1$ if $T_{j}<T_{i}$ and is otherwise 0, and $1_{\eta_{j}>\eta_{i}}=1$ if $\eta_{j}>\eta_{i}$. A C-index score equivalent to 1 corresponds to a perfect model estimator, while a C-index score of 0.5 represents a random estimator [42].

The C-index score can compare pairs in which the predictions and outputs are concordant, which means that the one with a higher risk score has a shorter actual survival time. If two instances experience an event at different times, or if one experiences an event and is outlasted by the other, we say that they are comparable. In contrast, a pair is said to not be comparable when they experience events at the same time [41,42].

#### 3.2.2. CoxNet (CN)

The Cox proportional hazard (CPH) assumes that the hazard is proportional to the instantaneous probability of an event at a particular time. In this case, the effect of the covariates is multiplying the hazard function by a function of the exploratory covariates. This means that two units of observation have a ratio of the constant of their hazards, and it depends on their covariate values [43].

Let $Xi=(X_{i1},\ldots ,X_{ip})$ be the realized values of the covariates for a subject *i*. The hazard function for the CPH model is described by [44]

(12) $$\lambda \left(t|X_{i}\right)=\lambda_{0}\left(t\right)\exp\left(\beta_{1}X_{i1}+\ldots +\beta_{p}X_{ip}\right)=\lambda_{0}\left(t\right)\exp\left(X_{i}\cdot \beta \right)$$

where $\lambda (t|X_{i})$ is the hazard function at time *t* for subject *i* with a covariate vector $X_{i}$, $\lambda_{0}\left(t\right)$ is the baseline hazard, and $\beta_{i}$ represents the effect parameters.

The CPH model is especially interpretive since the regression coefficients represent the hazard ratio, providing useful insights into the problem. However, in applications with a large set of features, the standard CPH fails due to the fact that the model convergence relies on inverting the matrix that becomes non-singular due to correlation among features [45].

The CoxNet (CN) overcomes these problems by implementing an Elastic Net regression with a weighted combination of the $l_{1}$ and $l_{2}$ penalty by solving [45]

(13) $$\arg\max_{\beta }\;\log PL\left(\beta \right)-\alpha \left(r\sum_{j=1}^{p}\left|\beta_{j}\right|+\frac{1-r}{2}\sum_{j=1}^{p}\beta_{j}^{2}\right)$$

where PL is the partial likelihood function of the Cox model, $\beta_{1},\ldots ,\beta_{p}$ are the coefficients for *p* features, α≥0 is a hyperparameter that controls the amount of shrinkage, and r∈[0;1[ is the relative weight of the l1 and l2 penalty. The $l_{1}$ penalty helps the model select only a subset of features, while $l_{2}$ leads to better stability through regularization. In this paper, we adopted the default value proposed for r=0.5 and an automatic procedure for selecting α≥0.01.

#### 3.2.3. Random Survival Forest (RSF)

**Figure 4:**
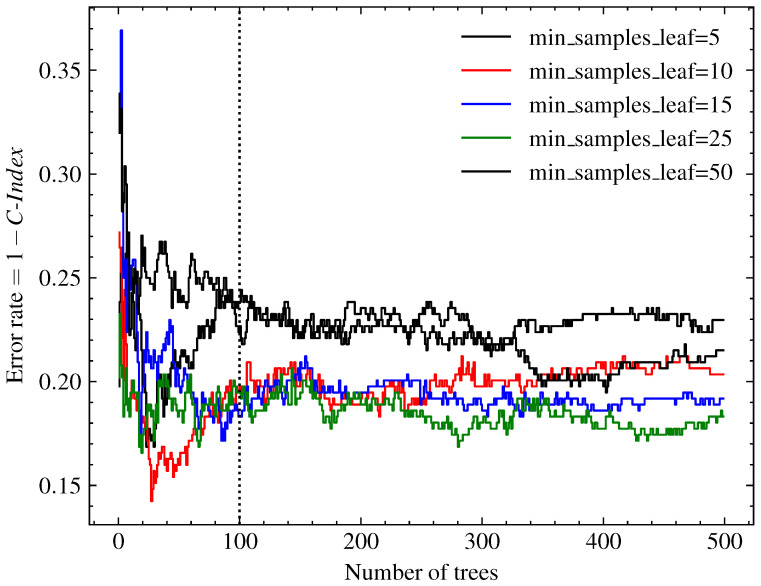
Analysis of the convergence of the RSF model, varying the parameters of the number of base estimators *M* and the minimum of samples in each node. We adopted a standard sample count of 15, since it achieved the lowest error for ensembles with M=100 estimators.

The random survival forest (RSF) model is an adaption of the random survival regressor for the analysis of right-censored survival data. The main components of the RSF algorithm are the growing of the survival trees and the forming of the ensemble cumulative hazard function. Survival trees are binary trees grown by the recursive splitting of tree nodes using a predetermined survival criterion. The splitting into nodes maximizes the survival distinction between the nodes, and eventually, each node of the tree becomes homogeneous and populated by cases with similar survival. Once training is complete, the cumulative hazard function estimation $\lambda (t|X_{i})$ of each survival tree is described by the function [46]

(14) $$\lambda \left(t|X_{i}\right)={\hat{\lambda }}_{h}\left(t\right)=\sum_{t_{l,h}\leq t}\frac{d_{l,h}}{Y_{l,h}}$$

where $d_{l,h}$ is the number of failures and $Y_{l,h}$ is the operation run at risk at time $t_{l,h}$. The ensemble cumulative function estimation $\lambda_{e}^{*}\left(t|X_{i}\right)$ is the simple average of the *M* base estimators and is given by [46]

(15) $$\lambda_{e}^{*}\left(t|X_{i}\right)=\frac{1}{M}\sum_{j=1}^{M}\lambda_{b}^{*}\left(t|X_{i}\right)$$

where $\lambda_{b}^{*}\left(t|X_{i}\right)$ represents the cumulative hazard function estimation of *j*th survival trees in the ensemble and *M* is the total number of survival trees. The base estimator hyperparameters were chosen from the convergence analysis performed on our data (shown in Figure 4). We used the parameter of the number of base estimators M=100, as it was a value close to the smallest error observed. For the minimum value of the samples in each node, the value adopted was 15 samples, selected because it presented the lowest error for ensembles with 100 trees.

#### 3.2.4. Gradient Boosting Survival Analysis (GBS)

The gradient boosting survival analysis (GBS) model was constructed using the gradient boosting framework for optimizing a specified loss function. The model was built on the principle of additively combining the predictions of multiple base learners into a powerful overall model [47]. GBS is an ensemble model similar to RSF, since it relies on multiple base learners to produce an overall prediction. The main difference between the two approaches is that while RSF independently fits the base learners and averages their predictions, the GBS model is assembled sequentially in a greedy, stage-wise manner. The GBS overall additive model *f* can be described by [47]

(16) $$f\left(X_{i}\right)=\sum_{m=1}^{M}\beta_{m}g\left(X_{i};\theta_{m}\right)$$

where M>0 represents the number of base learners, $\beta_{M}$ is the weighting term, the function *g* refers to a base learner outcome, and θ is the parameterized vector.

The loss function set for GBS is the partial likelihood loss of the CPH model. Therefore, the model maximizes the log partial likelihood function with the additive model f(X) such that [48]

(17) $$\arg\min_{f}\sum_{i=1}^{n}\delta_{i}\left[f\left(X_{i}\right)-\log\left(\sum_{j}\exp(f\left(X_{j}\right)\right)\right].$$

The base estimator of GBS, as in the RSF model, is the survival tree. In this way, we adopted the same control parameters for the estimator number *M* and a minimum number of samples in each node for both models.

### 3.3. Software and Hardware

All the routines, including data preparation, simulation, and result analysis, were developed using the Python language version 3.9.7 [49], adopting the following common scientific libraries: scipy 1.4.1 [50] for statistical analysis and hypothesis testing, pandas 1.2.4 [51] for data wrangling, numpy 1.20.2 [52] for array manipulation, scikit-learn 1.0.2 [53] for general data science functions, scikit-survival 0.17.2 [54] for survival model implementation, tsfresh 0.19.0 [37] for the TSF feature extraction model implementation, matplotlib 3.4.2 [55] and seaborn 0.11.2 [56] for plots and visualization, and eli5 0.13.0 for permutation importance testing.

The specifications of the hardware used to perform the simulation were as follows: CPU Intel Core i9 2.30 GHz, 16 GB of RAM installed, and the macOS v.12.5 operating system. The approximate amount of time necessary to perform the data preparation, feature selection, and all 100 simulations was around three hours (one hour for feature extraction and two hours for simulation) without any parallelization. All scripts are available from the researcher’s public repository (Github repository: https://github.com/rodrigosantis1/shp_prognosis accessed on 1 December 2022) for reproducibility and replicability. Data have not been made publicly available by the SHP but can be shared upon request.

## 4. Results and Discussion

### 4.1. Simulation Results

**Figure 5:**
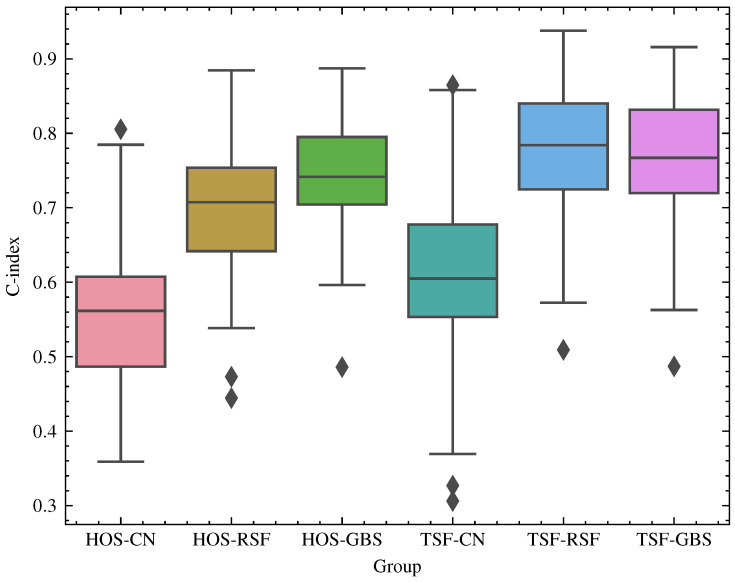
Box plot representation of the C-index scores by group of feature strategies and survival models for the 100 simulations performed. The groups with the highest accuracy were TSF-RSF and TSF-GBS, while the lowest was HOS-CN.

Figure 5 shows the C-index scores calculated for each of the 100 randomized simulations with different training and testing sets. This visualization format provides better understanding of the metric distribution of each of the CN, RSF, and GBS survival analysis models when combined with HOS and TSF feature engineering.

From the box plot analysis, we observed that the HOS-CN group obtained the lowest accuracy, while the TSF-RSF and TSF-GBS groups obtained the highest accuracies. The variance of CN was higher than those for the other survival models, especially when adopted with TSF feature engineering.

There were a few outliers in all models which were mostly in the lower bound, indicating possible convergence problems. A suggestion for both improving the variances and reducing outliers is to adopt a model selection schema for tuning and adjusting the models. The TSF-RSF and TSF-GBA groups presented close distribution in terms of both median and variance. In general, most of the RSF and GBS groups presented close variance.

Table 2 presents the average and standard deviation of the C-index score and fitting time, highlighting in bold the model with the highest score and the one with the lowest fitting time. The nonlinear models RSF and GSA, which require more computational time for training, achieved better accuracy scores than the linear model with regularization (CN). This trade-off between accuracy and computational time is expected in machine learning applications. When comparing RSF and GBS, RSF required up to 10 times more fitting time than GBS. However, it is worth mentioning that RSF, a bagging ensemble, can be more easily parallelized than GBS, a boosting ensemble. The fitting time difference between the TSF-CN and the nonlinear models was significant, requiring more than 1000 times less time than TSF-RSF for training.

Table 3 presents the total time necessary to execute both feature engineering strategies. As this is a step preceding model adjustment, it is worth considering its time when evaluating the models.

The computational time required to extract and select attributes using the TSF method was about 20 times greater than the time required using HOS. This is a significant difference that must be taken into account, especially for real-time applications of the prognosis model. However, it is interesting to point out that the TSF library offers the possibility of implementing cluster parallel computing. Furthermore, the time required for inference was lower, given that only the features previously selected by the feature hypothesis tests and applied in the model training needed to be calculated.

One-way ANOVA [57] was applied to test the null hypothesis that the groups had the same mean C-index score. Table 4 displays the $F_{S}$ statistics, which represent the ratio of the variance among score means divided by the average variance within groups, and the *p* value calculated for the statistics. By adopting a confidence level of 0.95, we rejected the hypothesis that the score was equal between all groups since the *p*-value was lower than alpha=0.05. Normality was checked using a Q-Q plot. The homogeneity of the variance when checking the ratio of the largest to the smallest sample standard deviations was less than 2 (1.44).

Sequentially, a pairwise Tukey test [58] was applied, and the results are presented in Table 5. The pair of groups in which the mean difference of the scores was not significant at the 0.95 level is highlighted in bold. The results show that the mean score metrics of the HOS-GBS, TSF-GBS, and TSF-RSF groups were statistically different. These results indicate that, from our experimentation, it is not possible to verify significant differences among the scores in these models. A reasonable model for the dataset we simulated was the HOS-GBS group, since it presented the least computational time for both preprocessing and fitting and was among the top three models.

### 4.2. Feature Importance Analysis

Feature importance was evaluated using the permutation importance method, which measures how the score decreases when a feature is not available [59]. The score adopted for evaluation was the C-index, the base estimator was the RSF model, and the number of permutation iterations was equal to 15.

Table 6 presents the 20 most important features of the HOS-RSF combination, detailed by the sensor and the statistic used for aggregation into the feature used to train the RSF model.

The features associated with the speed registered in the speed regulator and the temperature of the oil were the most important features, contributing to an average increase of 2.8% in the C-index score. The features related to the coupled side bearing temperature of the generator were the most frequent ones (4/20), the oil temperature of the hydraulic unit was the second-most-frequent feature (3/20), and the uncoupled side bearing temperature was the third-most-frequent feature (2/20). When analyzing the type of aggregation, kurtosis and variance were the most frequent types (6/20), and skewness and average were the least frequent types (4/20), although there was a balance among all four types.

Table 7 presents the top 20 most important features of the TSF-RSF combination, detailed by the sensor and the type of feature extraction technique applied.

The most important features were the CWT coefficient of the radial bushing temperature in the turbine and the absolute energy of the voltage in the bar, which contributed to an increase of 1.1% in the C-index score. Since more features were extracted using the TSF strategy than the HOS, it was expected that the weight of each individual feature would be lower. The features related to the radial bushing temperature in the turbine were most frequent (4/20), followed by those related to the coupled bearing temperature in the generator (2/20) and the bar voltage (2/20). The features originating from CWT were most frequent (9/20), followed by the FFT (3/20). The dominance of the CWT and FFT indicates the importance and efficiency of the time–frequency decomposition methods in this type of application.

It is important to note that the TSF algorithm includes the statistical aggregations of kurtosis, skewness, mean, and variance from the HOS feature engineering strategy. Additionally, none of these aggregations were present in the 20 most important attributes after the inclusion of more complex features, such as the FFT and CWT.

### 4.3. Model Application Analysis

In this section, we present a deeper look at the model which presented the highest mean score in the simulation: TSF-RSF. The C-index of the model was 0.8139. It is worth noting that the maximum value for the C-index is 1, which indicates the order of observed events followed the same order as all predicted events, and a C-index value of 0.5 indicates the prediction was no better than a random guess [32]. For comparative purposes, the application of the RSF method on the remaining service life of water mains obtained a C-index of 0.88 [32], while for modeling the disruption durations of a subway service, the metric was 0.672 [60].

**Figure 6:**
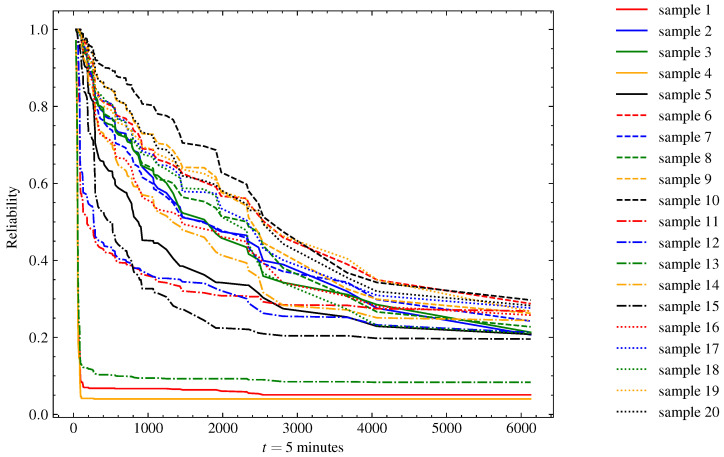
Reliability function estimate of test samples (*n* = 20) using TSF-RSF. With the passage of time units (t), the probability of the system not failing declines. According to the measured variables, the model estimates whether the reliability decays more abruptly or not. After 400 5 min time units, stability in the operation of the generating units is expected.

**Figure 7:**
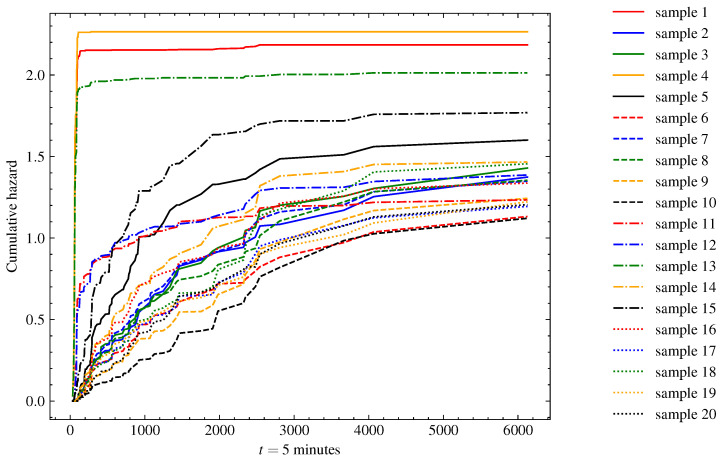
Cumulative hazard function estimate of test samples (*n* = 20) using TSF-RSF. The cumulative hazard risk increased as the operation time increased, with the highest rate occurring in the first 3000 5 min time units of operation. In unstable start-up operating cycles, this increase happened drastically in the first moments of operation.

Figure 6 presents the reliability, and Figure 7 presents the cumulative hazard function plots predicted by the model for 20 instances randomly selected from the test set. When analyzing the representations, we can identify three operation cycles with a reliability pitfall in the earliest minutes of operation. These indicate some cases in which there was an intrinsic problem in the generator-turbine system prior to or during start-up, and those systems must be stopped as soon as possible for maintenance. There was a second group of four instances in which the reliability dropped by half in the first 1000 5 min time units. This behavior might be related to some operating conditions that were observed in the operation of the machine. Finally, there was a third group containing the other instances with a steadier rhythm of reliability decay, in which more than half of the systems were expected to fail after 2000 5 min time units.

In practical applications, the survival model can be used to evaluate both the current and previous runs of a generator unit, returning both the risks and the expected remaining useful life. Maintenance teams may want to keep all their systems with a reliability function closer to the third group described before, especially right before the rainy periods. During these periods, the generation is higher, and the stopped periods are rarer, making it more difficult and less desirable to execute maintenance procedures on the machines, which may lead to a loss in power generation.

The model can also be extended for a prescriptive perspective combined with the parameters of the start-up process, aiming to optimize the start-up process in order to achieve the highest reliability level possible. With this, a longer lifetime of the assets and greater time between failures can be expected.

## 5. Conclusions

In the present paper, we presented a structured modeling pipeline for survival analysis and remaining useful life estimation in a small hydroelectric plant in CBM. The available period of operations was approximately 1 year, and the 54 variables were monitored in 4 generating units of the same model and manufacturer. The HOS-GBS, TSF-RSF, and TSF-GBS models presented the highest C-index scores in our simulation. All three are suitable for production deployment.

Identifying failures before they happen is crucial for allowing better management of asset maintenance, lowering operating costs, and in the case of SHPs, promoting the expansion of renewable energy sources in the energy matrix [61]. Applying time series feature engineering and machine learning survival models, such as a framework, aims to enhance the health of the equipment and decrease power generation downtime.

Looking at variable importance, variance and kurtosis represented the most frequent transformation functions in HOS feature engineering, while the FFT and CWT were the most frequent transformations in TSF feature engineering. The sensors that contributed the most to the model accuracy were the generator bearing temperature, hydraulic unit oil temperature, and turbine radial bushing temperature. The data-driven framework presents generalities, and thus it can be reused to model generator units with different types of sensors.

Future studies should examine feature and model selection through exhaustive searching and Bayesian or evolutionary optimization, as the parameters were manually adjusted. Fine-tuning the models can contribute even more to improving the model accuracy. From the point of the modeling assumptions, runs are set to be independent, but features can be crafted to include times from other runs and from the last imperfect or perfect repairs. Additionally, the predictive model opens a path for prescriptive optimization of the machine operation parameters, aiming to minimize wear, operational wear, and risk over time. Reinforcement learning approaches are a prominent course of action, since they have been adopted for dynamically developing maintenance policies for multi-component systems such as the power system of our object of study [62].

Finally, the present study contributes to the advancement of SHP maintenance, a crucial renewable power resource with enormous potential for supplying energy worldwide. By determining the faults before failure, management can carry out actions to avoid additional damage caused to combined systems and additional aggravation of the components, thus reducing the operating costs of power plants.

## Figures and Tables

**Table 1:** Descriptive information of the runs by generators contained in the dataset.

Generator	No. of Total Runs	No. of Faulty Runs	Avg. Cycle Time	Max. Cycle Time
1	133	50	691	4067
2	64	20	1270	6162
3	157	89	972	6835
4	130	40	764	3026

**Table 2:** C-index score and average computational time. TSF-RSF obtained the best average score, while HOS-CN achieved the lowest score at a reasonably lower fitting time.

Model	Description	C-Index	Fitting Time (s)
HOS-CN	Higher-Order Statistics + CoxNet	0.5562 ± 0.0898	0.0053 ± 0.0061
HOS-GBS	Higher-Order Statistics + Gradient Boosting Survival	0.7440 ± 0.0736	1.7514 ± 1.5064
HOS-RSF	Higher-Order Statistics + Random Survival Forest	0.7026 ± 0.0843	14.6232 ± 12.1850
TSF-CN	Tsfresh + CoxNet	0.6060 ± 0.1060	0.0193 ± 0.0023
TSF-GBS	Tsfresh + Gradient Boosting Survival	0.7644 ± 0.0854	9.7241 ± 2.2749
TSF-RSF	Tsfresh + Random Survival Forest	0.7744 ± 0.0903	27.5949 ± 23.2420

**Table 3:** Preprocessing time for feature engineering strategies. TSF technique requires about 20× more computational time than HOS.

Feature Engineering Strategy	Preprocessing Time (s)
Higher-Order Statistics (HOS)	4.68 s
Tsfresh (TSF)	67 s (extraction) + 26.6 s (selection)

**Table 4:** One-way ANOVA $F_{S}$ statistics and *P* value for the null hypothesis of scores being equal for all groups. The hypothesis $H_{0}:\mu_{1}=\mu_{2}=\mu_{3}=\ldots =\mu_{6}$ was rejected at the significance level of 1−α>0.95.

$F_{S}$ Statistics	*p* Value	Reject $H_{0}$
103.144	3.0036 × 10${}^{-78}$	True

**Table 5:** The pairwise Tukey test $q_{S}$ statistics and lower and upper bounds for mean differences between each pair of groups. The null hypothesis $H_{0}:\mu_{1}=\mu_{2}$ was rejected at a significance level of 1−α>0.95.

Group 1	Group 2	$\mu_{1}-\mu_{2}$	$q_{S}$ Statistics	Lower	Upper	Reject $H_{0}$
HOS-CN	HOS-GBS	0.1878	0.001	0.1519	0.2238	True
HOS-CN	HOS-RSF	0.1464	0.001	0.1105	0.1824	True
HOS-CN	TSF-CN	0.0498	0.0012	0.0139	0.0857	True
HOS-CN	TSF-GBS	0.2082	0.001	0.1723	0.2441	True
HOS-CN	TSF-RSF	0.2182	0.001	0.1823	0.2541	True
HOS-GBS	HOS-RSF	−0.0414	0.0132	−0.0773	−0.0055	True
HOS-GBS	TSF-CN	−0.138	0.001	−0.1739	−0.1021	True
HOS-GBS	TSF-GBS	0.0203	0.5743	−0.0156	0.0562	False
HOS-GBS	TSF-RSF	0.0304	0.152	−0.0056	0.0663	False
HOS-RSF	TSF-CN	−0.0966	0.001	−0.1325	−0.0607	True
HOS-RSF	TSF-GBS	0.0617	0.001	0.0258	0.0977	True
HOS-RSF	TSF-RSF	0.0718	0.001	0.0359	0.1077	True
TSF-CN	TSF-GBS	0.1584	0.001	0.1225	0.1943	True
TSF-CN	TSF-RSF	0.1684	0.001	0.1325	0.2043	True
TSF-GBS	TSF-RSF	0.01	0.9	−0.0259	0.0459	False

**Table 6:** The 20 most important features of HOS-RSF using permutation importance. Column weight represents the average increase in the C-index when the feature is available.

Sensor	Aggregation	Weight
Speed Regulator: Speed	Kurtosis	0.0285 ± 0.0346
Hydraulic Unit: Oil Temperature	Skewness	0.0281 ± 0.0156
Generator: Coupled Side Bearing Temperature	Skewness	0.0180 ± 0.0189
Turbine: Downstream Shaft Sealing Temperature	Average	0.0149 ± 0.0241
Voltage Regulator: Excitation Voltage	Variance	0.0120 ± 0.0120
Turbine: Radial Bushing Temperature	Average	0.0116 ± 0.0223
Spiral Case: Pressure	Kurtosis	0.0116 ± 0.0064
Generator: Current T	Variance	0.0114 ± 0.0083
Generator: Current S	Variance	0.0110 ± 0.0048
Speed Regulator: Distributor	Variance	0.0107 ± 0.0055
Generator: Coupled Side Bearing Temperature	Average	0.0103 ± 0.0092
Generator: Uncoupled Side Bearing Temperature	Skewness	0.0093 ± 0.0117
Coupled Side Bearing Vibration	Kurtosis	0.0091 ± 0.0127
Generator: Uncoupled Side Bearing Temperature	Kurtosis	0.0085 ± 0.0157
Generator: Voltage RN	Average	0.0081 ± 0.0141
Hydraulic Unit: Oil Temperature	Kurtosis	0.0079 ± 0.0096
Generator: Coupled Side Bearing Temperature	Kurtosis	0.0078 ± 0.0127
Hydraulic Unit: Flow Switch	Variance	0.0076 ± 0.0101
Hydraulic Unit: Oil Temperature	Variance	0.0074 ± 0.0085
Generator: Coupled Side Bearing Temperature	Skewness	0.0074 ± 0.0070

**Table 7:** The 20 most important features of the TSF-RSF group by permutation importance. Column weight represents the average increase in the C-index when the feature is available.

Sensor	Extraction	Weight
Turbine: Radial Bushing Temperature	CWT Coefficient	0.0122 ± 0.0211
Bar: Voltage	Abs. Energy	0.0112 ± 0.0256
Generator: Coupled Bearing Temperature	CWT Coefficient	0.0093 ± 0.0147
Speed Regulator: Speed	Energy Ratio	0.0083 ± 0.0144
Generator: Voltage RN	CWT Coefficient	0.0074 ± 0.0125
Generator: T-Phase Winding Temperature	Autocorrelation	0.0070 ± 0.0063
Turbine: Radial Bushing Temperature	CWT Coefficient	0.0066 ± 0.0204
Generator: Voltage TS	CWT Coefficient	0.0064 ± 0.0098
Generator: S-Phase Winding Temperature	CWT Coefficient	0.0064 ± 0.0044
Generator: Voltage ST	CWT Coefficient	0.0064 ± 0.0138
Generator: Reactive Power	Quantiles Change	0.0052 ± 0.0080
Bearing: Vertical Radial Vibration	Index Max Quantile	0.0052 ± 0.0044
Bar: Frequency	FFT Coefficient	0.0052 ± 0.0166
Turbine: Radial Bushing Temperature	Lempel Ziv Complexity	0.0052 ± 0.0064
Hydraulic Unit: Flow Switch	FFT Coefficient	0.0052 ± 0.0088
Bar: Voltage	CWT Coefficient	0.0052 ± 0.0182
Generator: Frequency	Quantiles Change	0.0050 ± 0.0054
Generator: Coupled Bearing Temperature	CWT Coefficient	0.0050 ± 0.0103
Turbine: Radial Bushing Temperature	Longest Strike above Mean	0.0050 ± 0.0172
Turbine: Downstream Shaft Sealing Temperature	FFT Coefficient	0.0050 ± 0.0078

## Data Availability

Data have not been made publicly available by the SHP but can be shared upon request.

## Abbreviations

The following abbreviations are used in this manuscript:

C-index	Concordance index
CN	CoxNet
CPH	Cox proportional hazard
CWT	Continous wavelet transform
DWT	Discrete Fourier transform
FFT	Fast Fourier transform
GBS	Gradient boosting survival analysis
HOS	Higher-order statistics
ICA	Independent component analysis
iForest	Isolation forest
PCA	Principal component analysis
RSF	Random survival forest
SHP	Small hydroelectric plants
TSF	Time series feature extraction on basis of scalable hypothesis tests

## References

[1] WEC (2019). World Energy Insights Brief.

[2] UNIDO (2016). World Small Hydropower Development Report 2016.

[3] Bousdekis A., Magoutas B., Apostolou D., Mentzas G. (2018). Review, analysis and synthesis of prognostic-based decision support methods for condition based maintenance. J. Intell. Manuf..

[4] Peng Y., Dong M., Zuo M.J. (2010). Current status of machine prognostics in condition-based maintenance: A review. Int. J. Adv. Manuf. Technol..

[5] Sikorska J.Z., Hodkiewicz M., Ma L. (2011). Prognostic modelling options for remaining useful life estimation by industry. Mech. Syst. Signal Process..

[6] Liu X., Kruger U., Littler T., Xie L., Wang S. (2009). Moving window kernel PCA for adaptive monitoring of nonlinear processes. Chemom. Intell. Lab. Syst..

[7] Žvokelj M., Zupan S., Prebil I. (2016). EEMD-based multiscale ICA method for slewing bearing fault detection and diagnosis. J. Sound Vib..

[8] Fu W., Wang K., Zhang C., Tan J. (2019). A hybrid approach for measuring the vibrational trend of hydroelectric unit with enhanced multi-scale chaotic series analysis and optimized least squares support vector machine. Trans. Inst. Meas. Control..

[9] Qiao L., Chen Q. (2015). Forecasting Models for Hydropower Unit Stability Using LS-SVM. Math. Probl. Eng..

[10] Vu V.H., Thomas M., Lafleur F., Marcouiller L. (2013). Towards an automatic spectral and modal identification from operational modal analysis. J. Sound Vib..

[11] Peng W.J., Luo X.Q., Guo P.C., Lu P. (2007). Vibration fault diagnosis of hydroelectric unit based on LS-SVM and information fusion technology. Zhongguo Dianji Gongcheng Xuebao/Proc. Chin. Soc. Electr. Eng..

[12] Gregg S.W., Steele J.P., Van Bossuyt D.L. (2017). Feature selection for monitoring erosive cavitation on a hydroturbine. Int. J. Progn. Health Manag..

[13] Ge Z., Song Z. (2007). Process monitoring based on independent Component Analysis-Principal Component Analysis (ICA-PCA) and similarity factors. Ind. Eng. Chem. Res..

[14] Zhu W., Zhou J., Xia X., Li C., Xiao J., Xiao H., Zhang X. (2014). A novel KICA-PCA fault detection model for condition process of hydroelectric generating unit. Meas. J. Int. Meas. Confed..

[15] de Souza Gomes A., Costa M.A., de Faria T.G.A., Caminhas W.M. (2013). Detection and classification of faults in power transmission lines using functional analysis and computational intelligence. IEEE Trans. Power Deliv..

[16] de Santis R.B., Costa M.A. (2020). Extended isolation forests for fault detection in small hydroelectric plants. Sustainability.

[17] Hara Y., Fukuyama Y., Arai K., Shimasaki Y., Osada Y., Murakami K., Iizaka T., Matsui T. Fault Detection of Hydroelectric Generators by Robust Random Cut Forest with Feature Selection Using Hilbert-Schmidt Independence Criterion. Proceedings of the 2021 IEEE International Conference on Smart Internet of Things (SmartIoT).

[18] Wu Y., Jiang B., Wang Y. (2020). Incipient winding fault detection and diagnosis for squirrel-cage induction motors equipped on CRH trains. ISA Trans..

[19] Si X.S., Wang W., Hu C.H., Zhou D.H. (2011). Remaining useful life estimation–a review on the statistical data driven approaches. Eur. J. Oper. Res..

[20] Salomon C.P., Ferreira C., Sant’Ana W.C., Lambert-Torres G., da Silva L.E.B., Bonaldi E.L., de Lacerda de Oliveira L.E., Torres B.S. (2019). A study of fault diagnosis based on electrical signature analysis for synchronous generators predictive maintenance in bulk electric systems. Energies.

[21] Lei Y., Li N., Guo L., Li N., Yan T., Lin J. (2018). Machinery health prognostics: A systematic review from data acquisition to RUL prediction. Mech. Syst. Signal Process..

[22] Zhao R., Yan R., Chen Z., Mao K., Wang P., Gao R.X. (2019). Deep learning and its applications to machine health monitoring. Mech. Syst. Signal Process..

[23] de Santis R.B., Gontijo T.S., Costa M.A. (2021). Condition-based maintenance in hydroelectric plants: A systematic literature review. Proc. Inst. Mech. Eng. Part J. Risk Reliab..

[24] An X., Pan L., Yang L. (2014). Condition parameter degradation assessment and prediction for hydropower units using Shepard surface and ITD. Trans. Inst. Meas. Control..

[25] Fu W., Wang K., Li C., Li X., Li Y., Zhong H. (2018). Vibration trend measurement for hydropower generator based on optimal variational mode decomposition and LSSVM improved with chaotic sine cosine algorithm optimization. Meas. Sci. Technol..

[26] Zhou K.B., Zhang J.Y., Shan Y., Ge M.F., Ge Z.Y., Cao G.N. (2019). A hybrid multi-objective optimization model for vibration tendency prediction of hydropower generators. Sensors.

[27] Dindorf C., Teufl W., Taetz B., Bleser G., Fröhlich M. (2020). Interpretability of input representations for gait classification in patients after total hip arthroplasty. Sensors.

[28] Tam I., Kalech M., Rokach L., Madar E., Bortman J., Klein R. (2020). Probability-based algorithm for bearing diagnosis with untrained spall sizes. Sensors.

[29] Khan I., Choi S., Kwon Y.W. (2020). Earthquake detection in a static and dynamic environment using supervised machine learning and a novel feature extraction method. Sensors.

[30] Voronov S., Krysander M., Frisk E. (2020). Predictive maintenance of lead-acid batteries with sparse vehicle operational data. Int. J. Progn. Health Manag..

[31] Gurung R.B. (2020). Random Forest for Histogram Data: An Application in Data-Driven Prognostic Models for Heavy-Duty Trucks. Ph.D. Thesis.

[32] Snider B., McBean E.A. (2021). Combining machine learning and survival statistics to predict remaining service life of watermains. J. Infrastruct. Syst..

[33] Mathur A., Cavanaugh K.F., Pattipati K.R., Willett P.K., Galie T.R. (2001). Reasoning and modeling systems in diagnosis and prognosis. Proceedings of the Component and Systems Diagnostics, Prognosis, and Health Management.

[34] de la Rosa J.J.G., Muñoz A.M. (2008). Higher-order cumulants and spectral kurtosis for early detection of subterranean termites. Mech. Syst. Signal Process..

[35] Nemer E., Goubran R., Mahmoud S. (2001). Robust voice activity detection using higher-order statistics in the LPC residual domain. IEEE Trans. Speech Audio Process..

[36] Welling M. Robust higher order statistics. Proceedings of the International Workshop on Artificial Intelligence and Statistics, PMLR.

[37] Christ M., Braun N., Neuffer J., Kempa-Liehr A.W. (2018). Time series feature extraction on basis of scalable hypothesis tests (tsfresh–a python package). Neurocomputing.

[38] Heckbert P. (1995). Fourier transforms and the fast Fourier transform (FFT) algorithm. Comput. Graph..

[39] Munoz A., Ertlé R., Unser M. (2002). Continuous wavelet transform with arbitrary scales and O (N) complexity. Signal Process..

[40] Attallah O., Karthikesalingam A., Holt P.J., Thompson M.M., Sayers R., Bown M.J., Choke E.C., Ma X. (2017). Using multiple classifiers for predicting the risk of endovascular aortic aneurysm repair re-intervention through hybrid feature selection. Proc. Inst. Mech. Eng. Part J. Eng. Med..

[41] Harrell Jr F.E., Lee K.L., Mark D.B. (1996). Multivariable prognostic models: Issues in developing models, evaluating assumptions and adequacy, and measuring and reducing errors. Stat. Med..

[42] Uno H., Cai T., Pencina M.J., D’Agostino R.B., Wei L.J. (2011). On the C-statistics for evaluating overall adequacy of risk prediction procedures with censored survival data. Stat. Med..

[43] Fisher L.D., Lin D.Y. (1999). Time-dependent covariates in the Cox proportional-hazards regression model. Annu. Rev. Public Health.

[44] Cox D.R. (1972). Regression models and life-tables. J. R. Stat. Soc. Ser..

[45] Simon N., Friedman J., Hastie T., Tibshirani R. (2011). Regularization paths for Cox’s proportional hazards model via coordinate descent. J. Stat. Softw..

[46] Ishwaran H., Kogalur U.B., Blackstone E.H., Lauer M.S. (2008). Random survival forests. Ann. Appl. Stat..

[47] Friedman J.H. (2002). Stochastic gradient boosting. Comput. Stat. Data Anal..

[48] Ridgeway G. (1999). The state of boosting. Comput. Sci. Stat..

[49] Van Rossum G., Drake F.L. (2009). Python 3 Reference Manual.

[50] Virtanen P., Gommers R., Oliphant T.E., Haberland M., Reddy T., Cournapeau D., Burovski E., Peterson P., Weckesser W., Bright J. (2020). SciPy 1.0: Fundamental Algorithms for Scientific Computing in Python. Nat. Methods.

[51] McKinney W. Data Structures for Statistical Computing in Python. Proceedings of the 9th Python in Science Conference.

[52] Harris C.R., Millman K.J., van der Walt S.J., Gommers R., Virtanen P., Cournapeau D., Wieser E., Taylor J., Berg S., Smith N.J. (2020). Array programming with NumPy. Nature.

[53] Pedregosa F., Varoquaux G., Gramfort A., Michel V., Thirion B., Grisel O., Blondel M., Prettenhofer P., Weiss R., Dubourg V. (2011). Scikit-learn: Machine Learning in Python. J. Mach. Learn. Res..

[54] Pölsterl S. (2020). scikit-survival: A Library for Time-to-Event Analysis Built on Top of scikit-learn. J. Mach. Learn. Res..

[55] Hunter J.D. (2007). Matplotlib: A 2D graphics environment. Comput. Sci. Eng..

[56] Waskom M.L. (2021). seaborn: Statistical data visualization. J. Open Source Softw..

[57] McDonald J. (2014). Handbook of Biological Statistics.

[58] Tukey J.W. (1977). Exploratory Data Analysis.

[59] Breiman L. (2001). Random forests. Mach. Learn..

[60] Wang X., Li J., Yu R. (2022). Modeling disruption durations of subway service via random survival forests: The case of Shanghai. J. Transp. Saf. Secur..

[61] Zhang Y., Zhao X., Zuo Y., Ren L., Wang L. (2017). The development of the renewable energy power industry under feed-in tariff and renewable portfolio standard: A case study of China’s photovoltaic power industry. Sustainability.

[62] Yousefi N., Tsianikas S., Coit D.W. (2020). Reinforcement learning for dynamic condition-based maintenance of a system with individually repairable components. Qual. Eng..

